# Neuronal c-Abl activation leads to induction of cell cycle and interferon signaling pathways

**DOI:** 10.1186/1742-2094-9-208

**Published:** 2012-08-31

**Authors:** Sarah D Schlatterer, Hyeon-sook Suh, Concepcion Conejero-Goldberg, Shufen Chen, Christopher M Acker, Sunhee C Lee, Peter Davies

**Affiliations:** 1 Department of Pathology, Albert Einstein College of Medicine, Bronx, NY, 10461, USA; 2 Litwin-Zucker Center for Research in Alzheimer’s Disease and Memory Disorders, Feinstein Center for Medical Research, North Shore, Long Island Jewish Health System, Manhasset, NY, 11030, USA

## Abstract

**Background:**

Expression of active c-Abl in adult mouse forebrain neurons in the AblPP/tTA mice resulted in severe neurodegeneration, particularly in the CA1 region of the hippocampus. Neuronal loss was preceded and accompanied by substantial microgliosis and astrocytosis. In contrast, expression of constitutively active Arg (Abl-related gene) in mouse forebrain neurons (ArgPP/tTA mice) caused no detectable neuronal loss or gliosis, although protein expression and kinase activity were at similar levels to those in the AblPP/tTA mice.

**Methods:**

To begin to elucidate the mechanism of c-Abl-induced neuronal loss and gliosis, gene expression analysis of AblPP/tTA mouse forebrain prior to development of overt pathology was performed. Selected results from gene expression studies were validated with quantitative reverse transcription PCR , immunoblotting and bromodeoxyuridine (BrdU) labeling, and by immunocytochemistry.

**Results:**

Two of the top pathways upregulated in AblPP/tTA mice with c-Abl expression for 2 weeks were cell cycle and interferon signaling. However, only the expression of interferon signaling pathway genes remained elevated at 4 weeks of c-Abl induction. BrdU incorporation studies confirm that, while the cell cycle pathway is upregulated in AblPP/tTA mice at 2 weeks of c-Abl induction, the anatomical localization of the pathway is not consistent with previous pathology seen in the AblPP/tTA mice. Increased expression and activation of STAT1, a known component of interferon signaling and interferon-induced neuronal excitotoxicity, is an early consequence of c-Abl activation in AblPP/tTA mice and occurs in the CA1 region of the hippocampus, the same region that goes on to develop severe neurodegenerative pathology and neuroinflammation. Interestingly, no upregulation of gene expression of interferons themselves was detected.

**Conclusions:**

Our data suggest that the interferon signaling pathway may play a role in the pathologic processes caused by c-Abl expression in neurons, and that the AblPP/tTA mouse may be an excellent model for studying sterile inflammation and the effects of interferon signaling in the brain.

## Introduction

The tyrosine kinase c-Abl has been shown to co-localize with tangles, plaques, and granulovacuolar degeneration in Alzheimer’s disease (AD) [1]. The c-Abl kinase also phosphorylates tau, the amyloid precursor protein (APP) and Fe65, an adaptor protein thought to play a role in APP processing [2-6]. The c-Abl tyrosine kinase has been shown to be activated by oxidative stress and treatment with Aβ peptides in neurons in culture [7]. These known activators of c-Abl are associated with aging and AD and, together with data showing c-Abl activation and co-localization with the characteristic lesions of AD, suggest that c-Abl may be activated during aging and neurodegenerative disease. The AblPP/tTA mouse model, which expresses an inducible, constitutively active form of c-Abl under the CamKIIα promoter using the Tet-Off system, was created to investigate the effects of c-Abl expression in adult forebrain neurons. The AblPP/tTA mouse develops progressive neurodegeneration and neuroinflammation in the CA1 region of the hippocampus, indicating that activation of c-Abl alone in adult neurons is sufficient to cause neuronal loss and inflammation [8].

Aberrant cell cycle activation has been shown to occur in neurons in human AD prior to neuronal death [9-13], and cell cycle events have been shown to precede amyloid deposition and microglial activation in several mouse models of AD [14]. Additionally, mice expressing a transgene that forces postmitotic neurons into the cell cycle recapitulate all the major pathological hallmarks of AD – neurodegeneration, neurofibrillary tangles, and amyloid plaques [15]. Chronic neuroinflammation and upregulation of a multitude of cytokines has been shown to occur in AD [16-26]. Others have suggested that stress/death signals produced by neurons may be the impetus for chronic inflammation in the brain [19,27].

Previously, the pathways that might be induced by c-Abl activity in neurons were unknown. In an effort to elucidate these pathways, we performed gene expression analysis on the forebrains of young AblPP/tTA mice at 2 or 4 weeks offf doxycycline. The expression of thousands of genes was altered in the AblPP/tTA mouse brain, and we chose to focus on two of the top pathways found to be upregulated in the AblPP/tTA mouse: cell cycle and interferon signaling. We show the anatomic location of the induction of cell cycle and interferon stimulated genes in the AblPP/tTA mouse brain, and that changes in interferon-stimulated gene expression occurred in neurons and co-localized in the CA1 region of the hippocampus, which was previously shown to develop severe neuronal loss and neuroinflammation. These data suggest that c-Abl expression in neurons can stimulate interferon signaling in the brain, and that this pathway may play a role in stimulating gliosis and promoting neuronal loss.

## Materials and methods

### Mice

Cohorts of five to six AblPP/tTA mice [8] and five to six wild-type littermates were used. Additional control mice carried either the CamKII-tTA gene alone or the c-Abl transgene alone. There was no elevation of c-Abl activity in these single transgenic mice. All mice were kept on a diet containing 200 mg/kg doxycycline (Bio-Serv Frenchtown, NJ 08825) throughout breeding, pregnancy, and weaning. Animals were housed in a facility with a 12-hour light/dark cycle and were provided with free access to water and food at all times. Mice were taken off doxycycline at approximately 4 weeks of age in order to induce c-Abl expression. Mice were euthanized with isofluorane and decapitated. Brains were hemisected sagitally. For all experiments, one half of the forebrain (randomly selected) was homogenized and frozen, while the other half was used for microarray analysis, quantitative reverse transcription PCR (qPCR), or immunohistochemistry.

### Gene expression analysis

Cohorts of six AblPP/tTA, six wild-type littermates, six c-Abl, and six CamKII-tTA mice at 2 or 4 weeks off doxycycline were used for gene expression analysis. The forebrain of one hemisphere was dissected away from hindbrain and frozen at −80°C prior to RNA extraction. Total RNA from mouse brain was extracted using standard protocols [28]. Total RNA was extracted with an RNeasy kit Valencia, CA 91355 , and RNA was measured for quality using an Agilent 2100 Bioanalyzer (Agilent Technologies, Palo Alto, CA, USA). Reverse transcription was performed using an ArrayScriptTM reverse transcriptase and T7-Oligo (dT)24 primers, followed by second-strand synthesis to generate double-stranded cDNA. The cDNA was converted to biotin-labeled cRNA (TotalprepTM RNA Labeling Kit, Ambion), then hybridized to the microarray platform. Mouse WG-6 Expression BeadChip (Illumina, San Diego, CA, USA) with 45,281 probes was the microarray platform used. Microarrays were utilized according to the manufacturer’s guidelines (Illumina).

Data was analyzed using the BeadStudio software (Illumina) and BRB Array Tools 3.8, a statistical software package designed for microarray analysis developed by the National Institutes of Health (linus.nci.nih.gov/BRB-ArrayTools.html). Expression differences were considered statistically significant if their *P* values were less than 0.001 using an F-test. Ingenuity Pathways Analysis application was used to determine which biological pathways were most upregulated in the AblPP/tTA mouse based on statistically significant changes in gene expression. The online database Interferome [29] was used to identify interferon signaling genes.

### Quantitative PCR

qPCR was performed according to standard protocols [30]. Briefly, total RNA extraction was performed using TRIzol reagent (Invitrogen) on hippocampus or cortex of cohorts of five AblPP/tTA mice and five wild-type controls at 2 weeks off doxycyline and 4 weeks off doxycycline according to manufacturer’s instructions. RNA was measured for quality using a NanoDrop. A SYBRGreen PCR mix was used to conduct qPCR with the ABI Prism 7900HT (Applied Biosystems). β-actin was used as an internal control, and each assay was performed in triplicate. Primers used for qPCR are listed in Table 1. The median value of the replicates for each sample was calculated and expressed as the cycle threshold (C_*T*_). ΔC_*T*_ was calculated as C_*T*_ of β-actin minus the C_*T*_ of the gene of interest in each sample. The relative amount of target gene expression in each sample was calculated as 2^ΔC*T*^, and fold change was calculated by dividing 2^ΔC*T*^ of the gene of interest by 2^ΔC*T*^ of the gene in the control sample. 

**Table 1:** Antibodies (IHC = immunohistochemistry, IHC-P = immunohistochemistry, paraffin sections; WB = immunoblot)

**Epitope**	**Antibody**	**Application**	**Source**
BrdU	1170376	IHC-P, 1:400	Roche
Cyclin B1	4135	WB, 1:1000	Cell Signaling Technology
Cyclin D1	2926	WB, 1:1000	Cell Signaling Technology
Cdk4	2906	WB, 1:1000	Cell Signaling Technology
Cdk2	2546	WB, 1:1000	Cell Signaling Technology
Polo-like kinase 1	05-844	WB, 1:1000	Millipore
Stat1	9172S	WB, 1:1000	Cell Signaling Technology
		IHC, 1:500	
Stat1 pY701	9167S	WB, 1:1000	Cell Signaling Technology
		IHC, 1:250	
Stat1 pS727	9177 s	WB, 1:1000	Cell Signaling Technology

BrdU, bromodeoxyuridine.

### Antibodies

Antibodies used for immunoblotting and immunohistochemistry are listed in Table 2.

**Table 2:** Primers

**Gene**	**Primer sequences**	**Source**
IFNα	F: GGATGTGACCTTCCTCAGACTC	OriGene
	R: ACCTTCTCCTGCGGGAATCCAA	
IFNβ	F: CAGCTCCAAGAAAGGACGAAC	qPrimerDepot
	R: GGCAGTGTAACTCTTCTGCAT	
IFNγ	F: GCGGCCTAGCTCTGAGACAA	Primer3
	R: GACTGTGCCGTGGCAGTAAC	
IL-6	F : ATGGATGCTACCAAACTGGAT	RTPrimerDB
	R : TGAAGGACTCTGGCTTTGTCT	
IP10	F : TCCTTGTCCTCCCTAGCTCA	RTPrimerDB
	R : ATAACCCCTTGGGAAGATGG	
IFIT3	F: GCTCAGGCTTACGTTGACAAGG	OriGene
	R: CTTTAGGCGTGTCCATCCTTCC	
IRF1	F: TCCAAGTCCAGCCGAGACACTA	OriGene
	R: ACTGCTGTGGTCATCAGGTAGG	
Isg15	F: CATCCTGGTGAGGAACGAAAGG	OriGene
	R: CTCAGCCAGAACTGGTCTTCGT	
PSMB8	F: CCTTACCTGCTTGGCACCATGT	OriGene
	R: TTGGATGCTGCAGACACGGAGA	
Stat1	F: GCCTCTCATTGTCACCGAAGAAC	OriGene
	R: TGGCTGACGTTGGAGATCACCA	
β-actin	F: TGCACCACCAACTGCTTAG	Primer3
	R: GGATGCAGGGATGATGTTC	

### Western blotting

Forebrain homogenates of AblPP/tTA mice and wild-type controls were used. Forebrain samples were homogenized in homogenization buffer (TBS with 10 mM NaF, 1 mM Na_3_VO_4_, 2 mM EGTA) with complete Mini protease inhibitors (Roche), frozen at −80°C, thawed, and spun at 14,000 rpm for 10 minutes at 4°C. Supernatants were analyzed in all experiments by immunoblotting using SDS-PAGE, according to standard protocols. Protein concentrations were normalized prior to use and all western blots were normalized to β-actin loading controls.

### Bromodeoxyuridine labeling

Bromodeoxyuridine (BrdU), 0.5 mg/ml, was administered to cohorts of three AblPP/tTA mice and three wild-type controls in drinking water for 5 days. Mice were euthanized immediately or allowed to age for 4 weeks. For detection of BrdU incorporation, one hemisphere of the brain was fixed overnight in 4% paraformaldehyde, embedded in paraffin, and sectioned in 5 μm increments. After rehydration, sections were steamed in 10 mM citrate buffer for 20 minutes. Endogenous peroxidase activity was blocked with 3% H_2_O_2_ for 10 minutes at room temperature. Sections were then treated with 3 N HCl for 30 minutes at room temperature and blocked in 5% goat serum/2% BSA for 1 hour. Primary antibody (anti-BrdU; Roche) was diluted in blocking solution and sections were incubated overnight at 4°C. Biotinylated secondary antibody was applied for 2 hours at room temperature, followed by streptavidin-HRP for 1 hour at room temperature. A solution of 20% Superblock (ThermoScientific) in TBS + 0.05% Triton X100 was used as a diluent for the secondary antibody and streptavidin-HRP. Staining was visualized with diaminobenzidine.

### Immunohistochemistry

Hemisected brains were fixed overnight in 4% paraformaldehyde and cut sagitally into 50 μm sections using a vibratome. Standard immunohistochemistry protocols were used. Endogenous peroxidase was blocked using 3% H_2_O_2_ in 0.25% TritonX 100 in TBS for 30 minutes at room temperature. Five percent milk/TBS was used as a blocking agent and primary antibody diluent. Sections were blocked for 1 hour at room temperature and incubated in primary antibody at 4°C overnight. After washing, biotinylated secondary antibody was applied for 2 hours at room temperature. Washes were repeated, and sections were incubated with Streptavidin-HRP for 1 hour at room temperature. A solution of 20% Superblock (Thermoscientific, Rockford, IL 61101) in TBS + 0.05% Triton X100 was used as a diluent for the secondary antibody and streptavidin-HRP. Staining was visualized with diaminobenzidine.

### Immunofluorescence

Endogenous peroxidase activity in vibratome sections of AblPP/tTA and wild-type control mouse brains was blocked by 3% H_2_O_2_ in 0.25% TritonX 100 in TBS for 30 minutes at room temperature. Blocking solution and primary antibody diluent was 5% milk. Sections were blocked for 1 hour at room temperature and incubated in primary antibodies at 4°C overnight. Biotinylated or fluorescently conjugated secondary antibody in 20% Superblock (ThermoScientific) in TBS + 0.05% TritonX100 was applied for 2 hours at room temperature. Sections were washed and incubated in Alexafluor conjugated streptavidin for 1 hour at room temperature and mounted on microscope slides. Secondary antibodies and streptavidin-HRP were diluted in 20% Superblock (ThermoScientific) in TBS + 0.05% TritonX100. Sections were incubated in 0.3% Sudan Black in 70% EtOH for 10 minutes at room temperature. Finally, slides were cover-slipped using Prolong® Gold anti-fade reagent with DAPI (Invitrogen). A Leica SP2 Scanning Laser Confocal Microscope was used to visualize fluorescence.

## Results

### Identification of two pathways induced by c-Abl activation in mouse forebrain

More than 6,000 genes were found to have significant changes in expression in the AblPP/tTA mice at 2 weeks off doxycyline versus wild-type controls, and more than 2,000 genes were found to have significant changes in expression in AblPP/tTA mice at 4 weeks off doxycyline. We used Ingenuity Pathways Analysis to determine the top pathways upregulated in AblPP/tTA mice versus wild-type mice at 2 and 4 weeks off doxycyline. Two of the top pathways that were shown to be upregulated in forebrains of AblPP/tTA mice were cell cycle and interferon signaling.

### Cell cycle activation in the AblPP/tTA mouse

**Figure 1:**
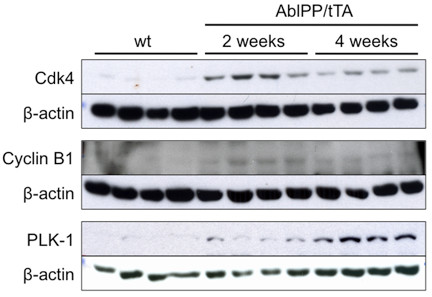
**Cell cycle activation in the AblPP/tTA mouse.** Western blotting of wild-type versus AblPP/tTA mouse forebrain homogenates. Cyclin B1 and Cdk4 levels are elevated in mice expressing consitutively active c-Abl for 2 weeks, but not 4 weeks, consistent with increased mRNA levels (Table 3). Polo-like kinase (PLK1) levels were elevated at 2 and 4 weeks, but mRNA levels were only elevated at 2 weeks.

**Figure 2:**
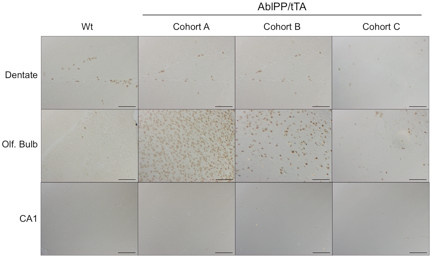
**Cell cycle activation occurs in the olfactory bulb but not in the CA1 region of the hippocampus in AblPP/tTA mice and decreases with time.** Bromodeoxyuridine (BrdU) labeling of wild-type versus AblPP/tTA mice. Cohort A = BrdU labeling at 2 weeks, euthanized at 2 weeks; Cohort B = BrdU labeling at 4 weeks, euthanized at 4 weeks; Cohort C = BrdU labeling at 2 weeks, euthanized at 6 weeks. Scalebars: Dentate, Olfactory bulb = 400 μm, CA1 = 800 μm.

Genes in the cell cycle pathway were strongly upregulated in AblPP/tTA mouse forebrain at 2 weeks off doxycyline, but no significant upregulation was present for any of the cell cycle-related genes at 4 weeks off doxycyline. Fold-change values of cell cycle genes in AblPP/tTA mice at 2 weeks off doxycycline are listed in Table 3. On immunoblot analysis, increases in polo-like kinase 1 (PLK-1), cyclin B1, and CDK 4 protein levels in forebrain homogenates at 2 weeks off doxycyline are apparent; however, levels of CDK 4 and cyclin B1 decrease at 4 weeks off doxycyline (Figure 1). Increases in PLK1 were evident on immunoblots at 4 weeks off doxicycline, but mRNA levels were not significantly elevated at this time point, as stated above. No changes in cyclin D1 or CDK 2 protein levels were observed (data not shown). Because immunoblot analysis could only be performed on whole forebrain homogenate, BrdU incorporation studies were undertaken in order to pinpoint which anatomical regions of the forebrain were experiencing cell cycle activation. Transgenic mice and controls were separated into three groups. Cohort A was administered BrdU for 5 days prior to euthanization at 2 weeks off doxycyline, cohort B was administered BrdU for 5 days prior to euthanization at 4 weeks off doxycyline, and cohort C was administered BrdU for 5 days at the same time off doxycyline as cohort A (beginning at about 1 week off doxycyline until 2 weeks off doxycyline) but allowed to age to 6 weeks off doxycyline. Surprisingly, no animals exhibited BrdU incorporation in the CA1 region of the hippocampus, where obvious neuronal loss occurs in AblPP/tTA mice. However, extensive labeling of the olfactory bulb occurred in mice in cohort A, with somewhat less labeling in cohort B, and the least amount of labeling occurred in cohort C (Figure 2). These results indicated that DNA replication, indicating cell cycle activation, is a very early and transient event in AblPP/tTA mice and seems to be limited to the olfactory bulb. The lack of BrdU labeling in cohort C indicates that the cells that undergo DNA replication in the early stages of c-Abl activation are lost with time. However, there does not appear to be an increase in DNA replication or expression of cell cycle proteins in pyramidal neurons in the CA1, as no cells in this region were found to be positive for BrdU, indicating that a separate process may be at work in that population of neurons.

**Table 3:** Cell cycle-related genes induced in AblPP/tTA mice at 2 weeks off doxycycline

**Fold-change**	**Unique ID**	**GeneBank Accession**	**Gene symbol**	**Description**
5.13	7200044	NM_007631	Ccnd1	Mus musculus cyclin D1 (Ccnd1), mRNA.
5.04	5690441	NM_007631	Ccnd1	Mus musculus cyclin D1 (Ccnd1), mRNA.
4.34	20364	NM_007631	Ccnd1	Mus musculus cyclin D1 (Ccnd1), mRNA.
4.03	670739	NM_175659	Hist1h2ah	Mus musculus histone cluster 1, H2ah (Hist1h2ah), mRNA.
4.03	3130609	NM_178183	Hist1h2ak	Mus musculus histone cluster 1, H2ak (Hist1h2ak), mRNA.
3.85	3520717	NM_178188	Hist1h2ad	Mus musculus histone cluster 1, H2ad (Hist1h2ad), mRNA.
3.85	1470341	NM_175659	Hist1h2ah	Mus musculus histone cluster 1, H2ah (Hist1h2ah), mRNA.
3.59	5490193	NM_178182	Hist1h2ai	Mus musculus histone cluster 1, H2ai (Hist1h2ai), mRNA.
3.58	4250711	NM_175661	Hist1h2af	Mus musculus histone cluster 1, H2af (Hist1h2af), mRNA.
3.46	6510253	NM_178185	Hist1h2ao	Mus musculus histone cluster 1, H2ao (Hist1h2ao), mRNA.
3.28	7200519	NM_007681	Cenpa	Mus musculus centromere protein A (Cenpa), mRNA.
3.27	7160253	NM_178188	Hist1h2ad	Mus musculus histone cluster 1, H2ad (Hist1h2ad), mRNA.
3.1	4610129	NM_178184	Hist1h2an	Mus musculus histone cluster 1, H2an (Hist1h2an), mRNA.
2.94	1580088	NM_009829	Ccnd2	Mus musculus cyclin D2 (Ccnd2), mRNA.
2.9	1260324	NM_009829	Ccnd2	Mus musculus cyclin D2 (Ccnd2), mRNA.
2.77	6060379	NM_007659	Cdc2a	Mus musculus cell division cycle 2 homolog A (S. pombe) (Cdc2a), mRNA.
2.77	1050170	NM_013538	Cdca3	Mus musculus cell division cycle associated 3 (Cdca3), mRNA.
2.76	4610722	NM_023223	Cdc20	Mus musculus cell division cycle 20 homolog (S. cerevisiae) (Cdc20), mRNA.
2.61	6450634	NM_026560	Cdca8	Mus musculus cell division cycle associated 8 (Cdca8), mRNA.
2.52	2190164	NM_172301	Ccnb1	Mus musculus cyclin B1 (Ccnb1), mRNA.
2.51	4250403	NM_011623	Top2a	Mus musculus topoisomerase (DNA) II alpha (Top2a), mRNA.
2.41	4390228	NM_023223	Cdc20	Mus musculus cell division cycle 20 homolog (S. cerevisiae) (Cdc20), mRNA.
2.34	630446	NM_183417	Cdk2	Mus musculus cyclin-dependent kinase 2 (Cdk2), transcript variant 1, mRNA.
2.33	520427	NM_011121	Plk1	Mus musculus polo-like kinase 1 (Drosophila) (Plk1), mRNA.
2.15	4570088	NM_023223	Cdc20	Mus musculus cell division cycle 20 homolog (S. cerevisiae) (Cdc20), mRNA.
2.12	1110390	NM_178182	Hist1h2ai	Mus musculus histone cluster 1, H2ai (Hist1h2ai), mRNA.
2.09	5820653	NM_175661	Hist1h2af	Mus musculus histone cluster 1, H2af (Hist1h2af), mRNA.
2.05	70546	NM_178186	Hist1h2ag	Mus musculus histone cluster 1, H2ag (Hist1h2ag), mRNA.
2.01	7550156	NM_172301	Ccnb1	Mus musculus cyclin B1 (Ccnb1), mRNA.
2.01	6860463	NM_009870	Cdk4	Mus musculus cyclin-dependent kinase 4 (Cdk4), mRNA.
1.77	1740039	NM_016756	Cdk2	Mus musculus cyclin-dependent kinase 2 (Cdk2), transcript variant 1, mRNA.
1.69	610717	NM_007632	Ccnd3	Mus musculus cyclin D3 (Ccnd3), transcript variant 1, mRNA.
1.61	1820274	NM_175384	Cdca2	Mus musculus cell division cycle associated 2 (Cdca2), mRNA.
1.6	1500446	NM_026560	Cdca8	Mus musculus cell division cycle associated 8 (Cdca8), mRNA.
1.58	4920148	NM_013538	Cdca3	Mus musculus cell division cycle associated 3 (Cdca3), mRNA.
1.46	6770286	AK077367	Ccnd2	Mus musculus cyclin D2 (Ccnd2), mRNA.
1.4	770162	NM_177733	E2f2	Mus musculus E2F transcription factor 2 (E2f2), mRNA.
1.37	1570754	NM_023223	Cdc20	Mus musculus cell division cycle 20 homolog (S. cerevisiae) (Cdc20), mRNA.
1.33	6550164	NM_177733	E2f2	Mus musculus E2F transcription factor 2 (E2f2), mRNA.
1.28	2000440	NM_183417	Cdk2	Mus musculus cyclin-dependent kinase 2 (Cdk2), transcript variant 1, mRNA.
1.26	4920468	NM_028023	Cdca4	Mus musculus cell division cycle associated 4 (Cdca4), mRNA.
1.24	7160066	NM_028023	Cdca4	Mus musculus cell division cycle associated 4 (Cdca4), mRNA.
1.23	3870112	NM_183417	Cdk2	Mus musculus cyclin-dependent kinase 2 (Cdk2), transcript variant 1, mRNA.

### Interferon signaling pathways in the AblPP/tTA mouse

**Figure 3:**
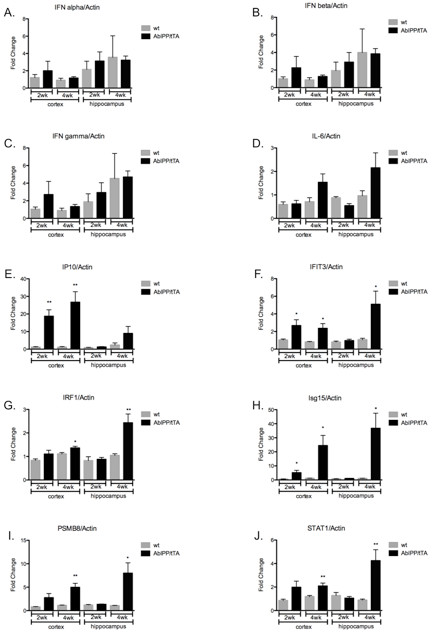
**Expression of interferon signaling genes is upregulated in AblPP/tTA mice, but expression of interferons themselves are not.** Quantitative real-time PCR of AblPP/tTA mouse cortex and hippocampus versus wild-type mouse cortex and hippocampus at 2 and 4 weeks off doxycyline. *P < 0.05, **P < 0.01.

Expression of interferon signaling genes was strongly upregulated in AblPP/tTA mouse forebrain at both 2 and 4 weeks off doxycyline. Table 4 lists fold change values for interferon signaling genes in AblPP/tTA mice. Interestingly, despite the strong induction of genes downstream of interferons α, β, and γ, there were no significant changes in gene expression of interferons themselves. In order to confirm these results, we selected six genes in the interferon signaling pathway and performed qPCR on the hippocampus and cortex of AblPP/tTA mice 2 and 4 weeks off doxycycline. The qPCR analysis revealed that there was strong upregulation of the six genes that were downstream of the interferons, but no significant upregulation of interferons themselves (Figure 3). IP10, IFIT3, IRF1, Isg15, PSMB8, and STAT1 were all significantly upregulated in AblPP/tTA mouse cortex at 4 weeks off doxycyline, and all these genes except IP10 were also significantly upregulated in the hippocampus. Despite the lack of statistical significance, IP10 does trend toward increased levels in the hippocampus at 4 weeks off doxycycline. The qPCR data indicate that the induction of interferon stimulated genes increases with time off doxycyline.

**Table 4:** Interferon related genes increased at 2 and 4 weeks off doxicycline

**Fold Change**		**Unique ID**	**GeneBank Accession**	**Gene symbol**	**Description**
**2 weeks**	**4 weeks**				
9.25	9.29	460221	XM_001472500	Isg15	PREDICTED: Mus musculus hypothetical protein LOC100038882 (LOC100038882), mRNA.
6.23	3.27	160463	NM_010708	Lgals3bp	Mus musculus lectin, galactoside-binding, soluble, 3 binding protein (Lgals3bp), mRNA.
5.59	2.19	3840292	NM_010509	Ifitm3	Mus musculus interferon induced transmembrane protein 3 (Ifitm3), mRNA.
5.07	3	2570095	NM_010724	Psmb8	Mus musculus proteasome (prosome, macropain) subunit, beta type 8 (large multifunctional peptidase 7) (Psmb8), mRNA.
5.05	2.56	1400041	NM_018734	Gbp2	Mus musculus guanylate binding protein 2 (Gbp2), mRNA.
5.02	4.24	1580528	NM_011909	Usp18	Mus musculus ubiquitin specific peptidase 18 (Usp18), mRNA.
4.89	3.63	7510020	NM_010501	Ifit3	Mus musculus interferon-induced protein with tetratricopeptide repeats 3 (Ifit3), mRNA.
4.8	3.08	6760390	NM_010387	H2-D1	Mus musculus histocompatibility 2, D region locus 1 (H2-D1), mRNA.
4.47	4.23	4830010	NM_011854	Oasl2	Mus musculus 2′-5′ oligoadenylate synthetase-like 2 (Oasl2), mRNA.
4.13	2.96	60553	NM_018734	Gbp3	Mus musculus guanylate nucleotide binding protein 3 (Gbp3), mRNA.
3.98	3.67	5270398	NM_008332	Ifit2	Mus musculus interferon-induced protein with tetratricopeptide repeats 2 (Ifit2), mRNA.
3.04	1.52	3940639	NM_027450	Glipr2	Mus musculus GLI pathogenesis-related 2 (Glipr2), mRNA.
2.96	1.62	4830543	NM_023065	Ifi27	Mus musculus interferon, alpha-inducible protein 27 (Ifi27), mRNA.
2.93	1.45	430167	NM_008534	Ly86	Mus musculus lymphocyte antigen 86 (Ly86), mRNA.
2.62	1.63	3140209	NM_013655	Cxcl10	Mus musculus chemokine (C-X-C motif) ligand 10 (Cxcl10), mRNA.
2.59	1.67	2230386	NM_008348	Iigp2	Mus musculus interferon inducible GTPase 2 (Iigp2), mRNA.
2.5	1.81	830762	NM_026030	Eif2ak2	Mus musculus eukaryotic translation initiation factor 2-alpha kinase 2 (Eif2ak2), mRNA.
2.47	1.94	6660634	NM_008390	Irf1	Mus musculus interferon regulatory factor 1 (Irf1), mRNA.
2.14	1.35	6660176	NM_213659	Stat3	Mus musculus signal transducer and activator of transcription 3 (Stat3), transcript variant 1, mRNA.
2.08	1.94	2000373	NM_009825	Serping1	Mus musculus serine (or cysteine) peptidase inhibitor, clade G, member 1 (Serping1), mRNA.
2.06	1.33	3780736	NM_001001892	H2-K1	Mus musculus histocompatibility 2, K1, K region (H2-K1), transcript variant 1, mRNA.
1.93	1.39	130598	NM_011852	Oas1g	Mus musculus 2′-5′ oligoadenylate synthetase 1 G (Oas1g), mRNA.
1.9	1.49	5720255	NM_201394	Pld4	Mus musculus phospholipase D family, member 4 (Pld4), mRNA.
1.86	1.43	4120014	NM_008330	Ifi35	Mus musculus interferon-induced protein 35 (Ifi35), mRNA.
1.85	1.48	4150050	NM_133779	Pigt	Mus musculus phosphatidylinositol glycan anchor biosynthesis, class T (Pigt), mRNA. XM_922599
1.84	1.87	3060482	NM_008394	Irf9	Mus musculus interferon regulatory factor 9 (Irf9), mRNA.
1.81	1.29	2100484	NM_025928	Plxnb2	PREDICTED: Mus musculus plexin B2, transcript variant 13 (Plxnb2), mRNA.
1.76	1.84	240725	NM_009283	Stat1	Mus musculus signal transducer and activator of transcription 1 (Stat1), mRNA.
1.72	2.11	520278	NM_024263	Mx2	Mus musculus myxovirus (influenza virus) resistance 2 (Mx2), mRNA.
1.67	1.4	5870093	NM_011530	Tap1	Mus musculus transporter 1, ATP-binding cassette, sub-family B (MDR/TAP) (Tap1), mRNA.
1.64	1.37	6770114	NM_001012236	Trem2	Mus musculus triggering receptor expressed on myeloid cells 2 (Trem2), mRNA.
1.61	1.29	3780279	NM_026116	Bax	Mus musculus Bcl2-associated X protein (Bax), mRNA.
1.55	1.32	5130139	NM_009369	Tgfb1	Mus musculus transforming growth factor, beta 1 (Tgfb1), mRNA.
1.55	1.22	1090139	NM_008332	Ifi47	Mus musculus interferon gamma inducible protein 47 (Ifi47), mRNA.
1.53	1.3	3060450	NM_011854	Oas2	Mus musculus 2′-5′ oligoadenylate synthetase 2 (Oas2), mRNA.
1.49	1.29	6590653	NM_008320	Irf7	Mus musculus interferon regulatory factor 7 (Irf7), mRNA.
1.47	1.49	5870398	NM_011970	Psmb10	Mus musculus proteasome (prosome, macropain) subunit, beta type 10 (Psmb10), mRNA.
1.45	1.25	460435	NM_016660	Hmg20b	Mus musculus high mobility group 20 B (Hmg20b), mRNA.
1.42	1.22	290494	XM_894155	BC004012	Mus musculus Nadk NAD kinase, mrNA.
1.4	1.42	6220594	NM_213659	Stat2	Mus musculus signal transducer and activator of transcription 2 (Stat2), mRNA.
1.4	1.37	4810072	NM_008490	Lcat	Mus musculus lecithin cholesterol acyltransferase (Lcat), mRNA.
1.39	1.42	2450554	NM_011175	Lgi4	Mus musculus leucine-rich repeat LGI family, member 4 (Lgi4), mRNA.
1.38	1.24	2480373	NM_026836	Taf10	Mus musculus TAF10 RNA polymerase II, TATA box binding protein (TBP)-associated factor (Taf10), mRNA.
1.33	1.39	6550376	NM_008529	Ly6e	Mus musculus lymphocyte antigen 6 complex, locus E (Ly6e), mRNA.
1.33	1.21	540411	NM_177663	Isg20	Mus musculus interferon-stimulated protein (Isg20), mRNA.
1.31	1.42	7210687	NM_007471	Apoe	Mus musculus apolipoprotein E (Apoe), mRNA.
1.26	1.22	990129	NM_026960	Grwd1	Mus musculus glutamate-rich WD repeat containing 1 (Grwd1), mRNA.
1.24	1.19	5870255	NM_028162	Tbc1d10a	Mus musculus TBC1 domain family, member 10a (Tbc1d10a), mRNA.
1.22	1.49	4890626	NM_008879	Lcn2	Mus musculus lipocalin 2 (Lcn2), mRNA.

**Figure 4:**
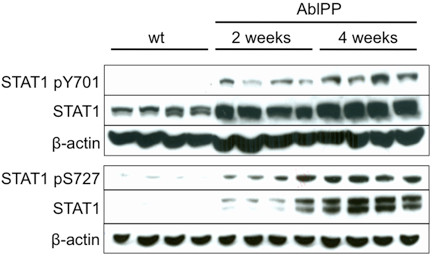
**Levels of activated STAT1 are increased in AblPP/tTA mice 2 and 4 weeks off doxycycline.** Western blot membranes were probed with antibodies to STAT1 pY701, STAT pS727, STAT1, and β-actin loading control. STAT1 pY701, STAT1 pS727, and STAT1 total protein levels were elevated in AblPP mice expressing constitutively active c-Abl for 2 and 4 weeks compared with wild-type controls.

We confirmed that STAT1, a key signaling molecule in the interferon pathway, is upregulated on a protein level in AblPP/tTA mouse forebrain by immunoblotting. Additionally, STAT1 expression is not only upregulated, but the protein itself is activated by phosphorylation at Y701 and Y727 (Figure 4). Phosphorylation of tyrosine 701 on STAT1 activates the protein, and phosphorylation of this site is necessary for STAT1 dimerization and translocation into the nucleus [31]. STAT1 signals remain high and seem to increase at 4 weeks off doxycyline, indicating that the interferon signaling pathway activation in AblPP/tTA mice is most likely not a transient event. 

### Interferon signaling is upregulated in neurons of the CA1 region of the hippocampus

**Figure 5:**
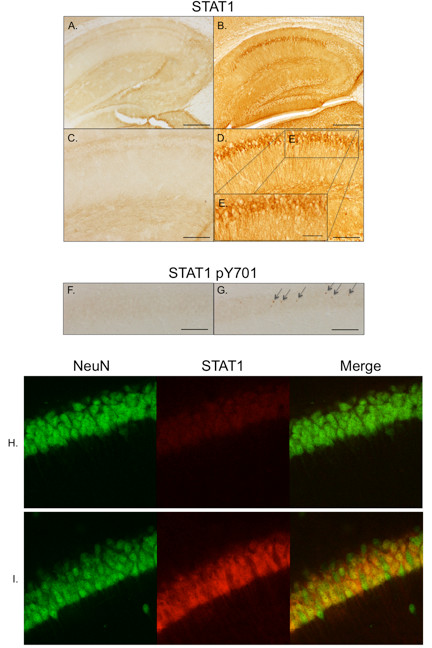
**STAT1 expression occurs in neurons of the CA1 region of the hippocampus in AblPP/tTA mice prior to neuronal loss. **(**A**-**E**) STAT1 immunohistochemistry of the CA1 region of an AblPP/tTA mouse expressing constitutively active c-Abl for 3 weeks (**B**,**D**,**E**) versus wild-type control (A,C). (**F**,**G**) STAT1pY701 immunohistochemistry the CA1 region of a wild-type mouse (**F**) versus an AblPP/tTA mouse 3 weeks off doxycyline (**G**). Cells positive for STAT1 pY701 are indicated with arrows. (**H**,**I**) Double labeling with NeuN (green) and total STAT1 (red) in wild-type (panel H) and AblPP mice expressing constitutively active c-Abl for 2 weeks (panel I). Scalebars: A,B = 2 mm; C,D,F,G = 800 μm; E = 400 μm.

In order to determine the cell type that demonstrates an increased expression of STAT 1, immunohistochemistry and double-labeling experiments were performed. In Figure 5, we show that STAT1 is elevated in neurons in the CA1 region of the hippocampus in AblPP/tTA mice, and that STAT1 co-localizes with the neuronal marker NeuN. These data indicate that STAT1 expression is induced in neurons themselves. Intriguingly, the STAT1 staining co-localizes to the CA1 region of the hippocampus, which experiences severe neuronal loss in aged AblPP/tTA mice.

## Discussion

It has been shown previously that c-Abl activation in adult neurons leads to neurodegeneration and neuroinflammation [8]. However, the mechanism by which c-Abl activation leads to these consequences remains unknown. In the work presented here, we show a correlation between c-Abl activation and the induction of two cell signaling pathways. First, transient but strong expression of cell cycle related proteins and DNA replication occurs in the olfactory bulb. Second, induction of interferon signaling genes occurs in the hippocampus, and the major signaling molecule of this pathway, STAT1, is upregulated in neurons in the CA1 region.

The first pathway examined was the cell cycle. The data show that expression of cell cycle related proteins and DNA replication does occur in AblPP/tTA mice, indicating that the cell cycle is activated, at least partially through S-phase. However, these findings do not localize to the CA1 region of the hippocampus. This suggests that aberrant cell cycle activation does not play a role in neuronal death observed in pyramidal neurons in CA1 of AblPP/tTA mice. Increased BrdU incorporation occurred mainly in the olfactory bulb, although BrdU-labeled cells were also found along a pathway anatomically consistent with the rostral migratory stream (data not shown). There is abundant expression of AblPP in the olfactory bulb [8].

Interestingly, BrdU labeling occurred in cells in the olfactory bulb when BrdU was administered at 2 weeks off doxycyline and mice were euthanized immediately, but BrdU labeling precipitously decreased when mice were administered BrdU at 2 weeks off doxycyline then aged for a further 4 weeks (Figure 2). Therefore, the cells that undergo DNA replication in the olfactory bulb of AblPP/tTA mice do not appear to survive for an extended period of time. The number of cells labeled in the dentate gyrus of AblPP/tTA mice also appears to decrease. This raises the possibility that c-Abl strongly induces DNA replication and possibly aberrant cell cycle activation at early time points, but that the cells that undergo this process may have impaired survival and decreased ability to replicate DNA as the mice age. BrdU labeling experiments performed on older AblPP/tTA mice support this hypothesis. In mice that were administered BrdU for 5 days prior to euthanization at 11 weeks off doxycyline, no labeling of the dentate gyrus occurred (Additional file 1: Figure S1).

The second pathway that we examined was interferon signaling. Our findings that interferon stimulated genes are induced in the presence of active c-Abl are consistent with previous reports showing that P210 BCR/Abl can induce expression of interferon responsive genes, including OAS1, IFIT1, IFI16, ISGF3G, and STAT1, in U937 cells in culture [32]. Also consistent with other studies, we find no evidence of significant induction of interferons themselves [32]. While other groups have focused on myeloid cells, this is the first evidence that interferon signaling pathways are induced by c-Abl activation in adult neurons *in vivo*. These data suggest that the AblPP/tTA mouse model will be useful to investigate the role of interferon signaling in neurodegeneration and neuroinflammation.

Given that STAT1, the major signaling molecule downstream of the interferons, was activated in the same anatomical region in which neurodegeneration occurs in AblPP/tTA mice, it is possible that STAT1 activation and interferon signaling play a role in c-Abl induced pathology in the CA1 region of the hippocampus. STAT1 activation has been associated with death in terminally differentiated cells and has been shown to play a role in apoptosis in response to TNFα treatment in cell culture [33]. Ischemia-reperfusion induced death is enhanced by STAT1 in cardiac myocytes, which, like neurons, are terminally differentiated [34,35]. Treatment of mice with paraquat, a toxin that induces selective loss of dopaminergic neurons, resulted in increased expression of STAT1 mRNA in the substantia nigra pars compact in a time course corresponding to dopaminergic neuronal loss [36]. Activated (pY701) STAT1 is upregulated in rat brain during focal cerebral ischemia-reperfusion experiments and localizes to the core of lesions, indicating that it may play a role in cell death during ischemia-reperfusion [37].

The role of other interferon signaling genes in neurodegenerative and neuroinflammatory diseases is less clear. Interferon signaling has been associated with cell loss, and, while we observe no significant increases in expression of interferons themselves, it is reasonable to assume that activation of downstream signaling molecules could have a similar effect as treatment with interferons themselves. In SHSY5Y cells, interferon β treatment results in apoptosis, and pretreatment of interferon β treated cells with P6, a pan-JAK inhibitor that results in downstream inhibition of STATs, led to decreased caspase 9, 7, and 3 cleavage and decreases in PARP expression, resulting in rescue of the apoptotic phenotype [38]. In primary neuronal cultures, long-term treatment with interferon β led to loss of neuritic processes and increased pY701 STAT1 and caspase 3 levels [38], and interferon γ knockout mice exhibited less neuronal loss in response to paraquat treatment, suggesting that interferon γ signaling may play a role in paraquat-induced neuronal loss [36]. Interferon regulatory factor 1 is thought to play a role in neuronal death in response to ischemia [39]. However, interferon regulatory factors 3 and 7 have been shown to be necessary for neuroprotection in multiple preconditioning paradigms for ischemia reperfusion injury [40].

While the precise role of cell cycle activation and interferon signaling in response to c-Abl activation in neurons remains unclear, we have demonstrated that c-Abl activation leads to an early, transient induction of cell cycle activation in the olfactory bulb and rostral migratory stream and to induction of interferon stimulated genes without (or prior to) induction of interferon expression. One interpretation of these data is that the cell cycle activation observed may be due to a transient stimulation of neurogenesis, although further studies would be necessary to confirm this hypothesis. Interestingly, there is evidence that interferon signaling downstream of c-Abl may have a role to play in stimulation of neurogenesis, as interferon treatment in 3xTg mice lead to increased neurogenesis in addition to increased severity of amyloid pathology and inflammation [41].

The localization of STAT1 activation in the CA1 region of the hippocampus, where severe neuronal loss and neuroinflammation occurs in aged AblPP/tTA mice, suggests that c-Abl induced expression of interferon signaling genes may play a role in the development of neuroinflammation and neurodegeneration observed in these mice. Further studies are necessary to determine whether STAT1 and other interferon signaling genes play a causative or protective role in neurodegeneration and neuroinflammation in AblPP/tTA mice. However, these data show a correlation between sites of neurodegeneration and gliosis and interferon signaling. Overall, these data show that the AblPP/tTA mouse may be an excellent model of sterile neuroinflammation in the context of neurodegeneration and could be used to elucidate the role of interferon signaling in neuroinflammatory and neurodegenerative disorders.

## Abbreviations

AD: Alzheimer’s disease; APP: amyloid precursor protein; Arg: Abl-related gene; BrdU: bromodeoxyuridine; PLK-1: polo-like kinase 1; qPCR: Quantitative reverse transcription polymerase chain reaction.

## Competing interests

The authors declare that they have no competing interests.

## Authors’ contributions

SD and PD designed these studies, and carried out all animal work and protein analyses. HS and SCL performed and analyzed the qPCR data. CG and SC performed and analyzed the microarray studies, and CA performed the immunohistochemisty. SD and PD wrote the manuscript, and all authors read and approved the final version.

## Supplementary Material

Additional file 1 **Figure S1.**Neurogenesis is apparently lost in AblPP/tTA mice with age. Bromodeoxyuridine (BrdU) labeling of the dentate gyrus of wild-type versus AblPP/tTA mice at 11 weeks off doxycyline. Scalebars = 400 μm. The localization of BrdU labeled cells is consistent with the distribution of neuroblasts. Click here for file

## References

[B1] Jing Z Caltagarone J Bowser R Altered subcellular distribution of c-Abl in Alzheimer’s disease J Alzheimers Dis 2009 17 409 422 19363261 10.3233/JAD-2009-1062 PMC2829466

[B2] Perkinton MS Standen CL Lau KF Kesavapany S Byers HL Ward M McLoughlin DM Miller CC The c-Abl tyrosine kinase phosphorylates the Fe65 adaptor protein to stimulate Fe65/amyloid precursor protein nuclear signaling J Biol Chem 2004 279 22084 22091 10.1074/jbc.M311479200 15031292

[B3] Vazquez MC Vargas LM Inestrosa NC Alvarez AR c-Abl modulates AICD dependent cellular responses: transcriptional induction and apoptosis J Cell Physiol 2009 220 136 143 10.1002/jcp.21743 19306298

[B4] Zambrano N Bruni P Minopoli G Mosca R Molino D Russo C Schettini G Sudol M Russo T The beta-amyloid precursor protein APP is tyrosine-phosphorylated in cells expressing a constitutively active form of the Abl protoncogene J Biol Chem 2001 276 19787 19792 10.1074/jbc.M100792200 11279131

[B5] Tremblay MA Acker CM Davies P Tau phosphorylated at tyrosine 394 is found in Alzheimer’s disease tangles and can be a product of the Abl-related kinase, Arg J Alzheimers Dis 2010 19 721 733 20110615 10.3233/JAD-2010-1271 PMC2949685

[B6] Derkinderen P Scales TM Hanger DP Leung KY Byers HL Ward MA Lenz C Price C Bird IN Perera T Kellie S Williamson R Noble W Van Etten RA Leroy K Brion JP Reynolds CH Anderton BH Tyrosine 394 is phosphorylated in Alzheimer’s paired helical filament tau and in fetal tau with c-Abl as the candidate tyrosine kinase J Neurosci 2005 25 6584 6593 10.1523/JNEUROSCI.1487-05.2005 16014719 PMC6725430

[B7] Alvarez AR Sandoval PC Leal NR Castro PU Kosik KS Activation of the neuronal c-Abl tyrosine kinase by amyloid-beta-peptide and reactive oxygen species Neurobiol Dis 2004 17 326 336 10.1016/j.nbd.2004.06.007 15474370

[B8] Schlatterer SD Tremblay MA Acker CM Davies P Neuronal c-Abl overexpression leads to neuronal loss and neuroinflammation in the mouse forebrain J Alzheimers Dis 2011 25 119 133 21368377 10.3233/JAD-2011-102025 PMC3349237

[B9] Vincent I Jicha G Rosado M Dickson DW Aberrant expression of mitotic cdc2/cyclin B1 kinase in degenerating neurons of Alzheimer’s disease brain J Neurosci 1997 17 3588 3598 9133382 10.1523/JNEUROSCI.17-10-03588.1997 PMC6573674

[B10] Busser J Geldmacher DS Herrup K Ectopic cell cycle proteins predict the sites of neuronal cell death in Alzheimer’s disease brain J Neurosci 1998 18 2801 2807 9525997 10.1523/JNEUROSCI.18-08-02801.1998 PMC6792587

[B11] Vincent I Rosado M Davies P Mitotic mechanisms in Alzheimer’s disease? J Cell Biol 1996 132 413 425 10.1083/jcb.132.3.413 8636218 PMC2120731

[B12] Yang Y Geldmacher DS Herrup K DNA replication precedes neuronal cell death in Alzheimer’s disease J Neurosci 2001 21 2661 2668 11306619 10.1523/JNEUROSCI.21-08-02661.2001 PMC6762514

[B13] Yang Y Mufson EJ Herrup K Neuronal cell death is preceded by cell cycle events at all stages of Alzheimer’s disease J Neurosci 2003 23 2557 2563 12684440 10.1523/JNEUROSCI.23-07-02557.2003 PMC6742098

[B14] Yang Y Varvel NH Lamb BT Herrup K Ectopic cell cycle events link human Alzheimer’s disease and amyloid precursor protein transgenic mouse models J Neurosci 2006 26 775 784 10.1523/JNEUROSCI.3707-05.2006 16421297 PMC6675370

[B15] Park KH Hallows JL Chakrabarty P Davies P Vincent I Conditional neuronal simian virus 40 T antigen expression induces Alzheimer-like tau and amyloid pathology in mice J Neurosci 2007 27 2969 2978 10.1523/JNEUROSCI.0186-07.2007 17360920 PMC6672567

[B16] McGeer PL McGeer EG The inflammatory response system of brain: implications for therapy of Alzheimer and other neurodegenerative diseases Brain Res Brain Res Rev 1995 21 195 218 8866675 10.1016/0165-0173(95)00011-9

[B17] Akiyama H Barger S Barnum S Bradt B Bauer J Cole GM Cooper NR Eikelenboom P Emmerling M Fiebich BL Finch CE Frautschy S Griffin WS Hampel H Hull M Landreth G Lue L Mrak R Mackenzie IR McGeer PL O’Banion MK Pachter J Pasinetti G Plata-Salaman C Rogers J Rydel R Shen Y Streit W Strohmeyer R Tooyoma I Inflammation and Alzheimer’s disease Neurobiol Aging 2000 21 383 421 10.1016/S0197-4580(00)00124-X 10858586 PMC3887148

[B18] Bamberger ME Landreth GE Inflammation, apoptosis, and Alzheimer’s disease Neuroscientist 2002 8 276 283 12061507 10.1177/1073858402008003013

[B19] Wyss-Coray T Mucke L Inflammation in neurodegenerative disease–a double-edged sword Neuron 2002 35 419 432 10.1016/S0896-6273(02)00794-8 12165466

[B20] Wyss-Coray T Inflammation in Alzheimer disease: driving force, bystander or beneficial response? Nat Med 2006 12 1005 1015 16960575 10.1038/nm1484

[B21] Glass CK Saijo K Winner B Marchetto MC Gage FH Mechanisms underlying inflammation in neurodegeneration Cell 2010 140 918 934 10.1016/j.cell.2010.02.016 20303880 PMC2873093

[B22] Griffin WS Sheng JG Royston MC Gentleman SM McKenzie JE Graham DI Roberts GW Mrak RE Glial-neuronal interactions in Alzheimer’s disease: the potential role of a ‘cytokine cycle’ in disease progression Brain Pathol 1998 8 65 72 9458167 10.1111/j.1750-3639.1998.tb00136.x PMC8098321

[B23] Lukiw WJ Gene expression profiling in fetal, aged, and Alzheimer hippocampus: a continuum of stress-related signaling Neurochem Res 2004 29 1287 1297 15176485 10.1023/b:nere.0000023615.89699.63

[B24] Griffin WS Sheng JG Roberts GW Mrak RE Interleukin-1 expression in different plaque types in Alzheimer’s disease: significance in plaque evolution J Neuropathol Exp Neurol 1995 54 276 281 10.1097/00005072-199503000-00014 7876895

[B25] Sheng JG Mrak RE Griffin WS Glial-neuronal interactions in Alzheimer disease: progressive association of IL-1alpha + microglia and S100beta + astrocytes with neurofibrillary tangle stages J Neuropathol Exp Neurol 1997 56 285 290 10.1097/00005072-199703000-00007 9056542

[B26] Tarkowski E Blennow K Wallin A Tarkowski A Intracerebral production of tumor necrosis factor-alpha, a local neuroprotective agent, in Alzheimer disease and vascular dementia J Clin Immunol 1999 19 223 230 10.1023/A:1020568013953 10471976

[B27] Aldskogius H Liu L Svensson M Glial responses to synaptic damage and plasticity J Neurosci Res 1999 58 33 41 10.1002/(SICI)1097-4547(19991001)58:1<33::AID-JNR5>3.0.CO;2-M 10491570

[B28] Conejero-Goldberg C Hyde TM Chen S Dreses-Werringloer U Herman MM Kleinman JE Davies P Goldberg TE Molecular signatures in post-mortem brain tissue of younger individuals at high risk for Alzheimer’s disease as based on APOE genotype Mol Psychiatry 2011 16 836 847 10.1038/mp.2010.57 20479757 PMC2953572

[B29] Samarajiwa SA Forster S Auchettl K Hertzog PJ INTERFEROME: the database of interferon regulated genes Nucleic Acids Research 2009 Jan 37 Database Issue D852 D857 18996892 10.1093/nar/gkn732 PMC2686605

[B30] Suh HS Cosenza-Nashat M Choi N Zhao ML Li JF Pollard JW Jirtle RL Goldstein H Lee SC Insulin-like growth factor 2 receptor is an IFNgamma-inducible microglial protein that facilitates intracellular HIV replication: implications for HIV-induced neurocognitive disorders Am J Pathol 2010 177 2446 2458 10.2353/ajpath.2010.100399 20889566 PMC2966802

[B31] Darnell JE Kerr IM Stark GR Jak-STAT pathways and transcriptional activation in response to IFNs and other extracellular signaling proteins Science 1994 264 1415 1421 10.1126/science.8197455 8197455

[B32] Hakansson P Segal D Lassen C Gullberg U Morse HC Fioretos T Meltzer PS Identification of genes differentially regulated by the P210 BCR/ABL1 fusion oncogene using cDNA microarrays Exp Hematol 2004 32 476 482 10.1016/j.exphem.2004.02.012 15145216

[B33] Kumar A Commane M Flickinger TW Horvath CM Stark GR Defective TNF-alpha-induced apoptosis in STAT1-null cells due to low constitutive levels of caspases Science 1997 278 1630 1632 10.1126/science.278.5343.1630 9374464

[B34] Stephanou A Brar BK Scarabelli TM Jonassen AK Yellon DM Marber MS Knight RA Latchman DS Ischemia-induced STAT-1 expression and activation play a critical role in cardiomyocyte apoptosis J Biol Chem 2000 275 10002 10008 10.1074/jbc.275.14.10002 10744676

[B35] Stephanou A Scarabelli TM Brar BK Nakanishi Y Matsumura M Knight RA Latchman DS Induction of apoptosis and Fas receptor/Fas ligand expression by ischemia/reperfusion in cardiac myocytes requires serine 727 of the STAT-1 transcription factor but not tyrosine 701 J Biol Chem 2001 276 28340 28347 10.1074/jbc.M101177200 11309387

[B36] Mangano EN Litteljohn D So R Nelson E Peters S Bethune C Bobyn J Hayley S Interferon-gamma plays a role in paraquat-induced neurodegeneration involving oxidative and proinflammatory pathways Neurobiol Aging 2011 33 1411 1426 21482445 10.1016/j.neurobiolaging.2011.02.016

[B37] West DA Valentim LM Lythgoe MF Stephanou A Proctor E van der Weerd L Ordidge RJ Latchman DS Gadian DG MR image-guided investigation of regional signal transducers and activators of transcription-1 activation in a rat model of focal cerebral ischemia Neuroscience 2004 127 333 339 10.1016/j.neuroscience.2004.05.022 15262323

[B38] Dedoni S Olianas MC Onali P Interferon-beta induces apoptosis in human SH-SY5Y neuroblastoma cells through activation of JAK-STAT signaling and down-regulation of PI3K/Akt pathway J Neurochem 2010 115 1421 1433 10.1111/j.1471-4159.2010.07046.x 21044071

[B39] Alexander M Forster C Sugimoto K Clark HB Vogel S Ross ME Iadecola C Interferon regulatory factor-1 immunoreactivity in neurons and inflammatory cells following ischemic stroke in rodents and humans Acta Neuropathol 2003 105 420 424 12677441 10.1007/s00401-002-0658-x

[B40] Stevens SL Leung PY Vartanian KB Gopalan B Yang T Simon RP Stenzel-Poore MP Multiple preconditioning paradigms converge on interferon regulatory factor-dependent signaling to promote tolerance to ischemic brain injury J Neurosci 2011 31 8456 8463 10.1523/JNEUROSCI.0821-11.2011 21653850 PMC3130521

[B41] Mastrangelo MA Sudol KL Narrow WC Bowers WJ Interferon-{gamma} differentially affects Alzheimer’s disease pathologies and induces neurogenesis in triple transgenic-AD mice Am J Pathol 2009 175 2076 2088 10.2353/ajpath.2009.090059 19808651 PMC2774071

