# Supplementary figures and images for: Neuronal c-Abl activation leads to induction of cell cycle and interferon signaling pathways
Source: J Neuroinflammation. 2012 Aug 31;9:208. doi: 10.1186/1742-2094-9-208 (PMC3488571; doi:10.1186/1742-2094-9-208)

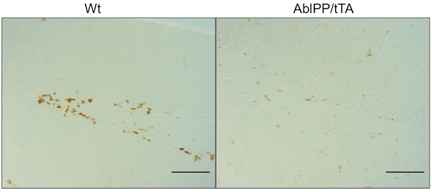

Supplement: Additional file 1 — Figure S1.Neurogenesis is apparently lost in AblPP/tTA mice with age. Bromodeoxyuridine (BrdU) labeling of the dentate gyrus of wild-type versus AblPP/tTA mice at 11 weeks off doxycyline. Scalebars = 400 μm. The localization of BrdU labeled cells is consistent with the distribution of neuroblasts. [file 1742-2094-9-208-S1.tiff]
